# Molecular mechanisms and computational insights into human SGLTs: advancing toward selective SGLT1 inhibition

**DOI:** 10.3389/fmolb.2025.1668400

**Published:** 2025-10-29

**Authors:** Nadhiri Kaijage, Sebastian Kraszewski

**Affiliations:** Department of Biomedical Engineering, Faculty of Fundamental Problems of Technology, Wroclaw University of Science and Technology, Wroclaw, Poland

**Keywords:** sodium-glucose cotransporter 1 (SGLT1), sodium-glucose cotransporter 2 (SGLT2), selective SGLT1 inhibition, glucose transport mechanisms, cryo-electron microscopy (cryo-EM), molecular dynamics simulations

## Abstract

The sodium and glucose transporters (SGLTs) are integral membrane proteins crucial for glucose homeostasis, with SGLT1 and SGLT2 being widely studied as primary therapeutic targets. Despite SGLT2 inhibitors having been well clinically established, selective SGLT1 inhibition remains an unmet goal, although its potential in managing diabetes, cardiovascular disease, and cancer. Recent advances in structural biology, including cryo-electron microscopy and computational modeling approaches, have provided significant avenues into the molecular mechanisms of SGLTs and their inhibition. High-resolution structural data now reveal inhibitor binding modes and conformational dynamics, while molecular dynamics simulations, free energy calculations, and AlphaFold2 predictions further explain sodium coupling and conformational transitions. Notable differences between SGLT1 and SGLT2 include selectivity determinants, ${Na}^{+}$ site occupancy, and gating mechanisms, which inform drug design but also pose challenges for achieving SGLT1 specificity. Homology modeling and MD simulations, strongly validated by cryo-EM, mutagenesis, and uptake/binding assays, are complemented by binding free energy calculations and 3D-RISM hydration analysis, with rising use of AlphaFold predicted models tied to experimental maps; key open questions include the absence of Na3 density in SGLT2, isoform-specific MAP17 dependence, and how differences in the central binding cavity of SGLT1 versus SGLT2 can be leveraged for selectivity. Integrating advanced computational approaches, including Artificial Intelligence and Machine Learning, offers promising avenues to explore inhibitor-induced conformational changes and advance the rational design of selective SGLT1 inhibitors. This review proposes a new framework for selective SGLT1 inhibitor development by aligning computational predictions with experimental validations.

## Introduction to SGLTs

Glucose is the center of mammals’ metabolism, providing energy for cellular processes and active transport (Nakrani et al., 2020). The sodium and glucose transporters (SGLTs) represent a family of membrane proteins that facilitate glucose transport across cellular membranes; they are secondary active transporters utilizing the sodium ion gradient established by ${Na}^{+}$ / $K^{+}$ ATPase as a driving force. SGLTs are integral membrane proteins that belong to the SLC5A family of solute carriers, comprising at least six isoforms with distinct substrate selectivities (Kamitori et al., 2022). They operate via an alternating-access mechanism, switching between multiple states to deliver cargo from the external membrane side to the cytoplasm (Wright et al., 2011; Bisignano et al., 2020). In the alternating-access kinetic model, SGLTs cycle through 6 conformational states (C1-C6), conformational transitions occur at a time constant in the range of 2–20 ms, and binding of 3–30 ms (Sal et al., 2012). However, the exact nature of the conformational transition and the energetic details that govern the transport cycle remain unclear. SGLTs harness the inward ${Na}^{+}$ electrochemical gradient to drive uphill glucose uptake, enabling cells to accumulate glucose even against its concentration gradient (Sano et al., 2020).

**FIGURE 1:**
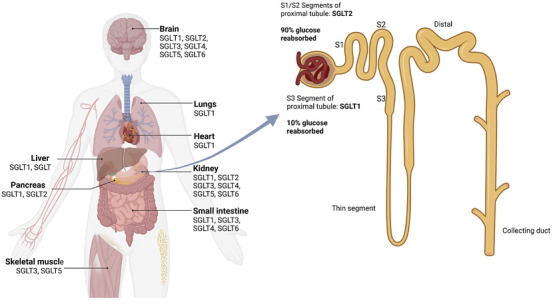
Tissue distribution and renal segment localization of human SGLT isoforms. Left, organs annotated with reported expression of SGLT1–SGLT6. Right, nephron schematic showing early proximal-tubule segments S1/S2 (apical SGLT2) that reabsorb ≈ 90% of filtered glucose and the S3 segment (apical SGLT1) that reclaims the remaining ≈ 10% (values refer to euglycemia).

SGLT1 and SGLT2 are the most extensively studied for glucose homeostasis (Sano et al., 2020). SGLT1 is primarily expressed in the small intestine and in the S3 segment of the renal proximal tubule Figure 1, where it plays a critical role in the absorption of glucose and galactose from the intestinal lumen and the reabsorption of glucose from the renal filtrate, respectively (Wright et al., 2011). In contrast, SGLT2 is primarily localized in the S1 and S2 segments of the renal proximal tubule Figure 1 (Uchida et al., 2015), where it is responsible for the majority of glucose reuptake of 80–90 percent of filtered glucose (Abdul-Ghani et al., 2015), thus preventing glucose loss in urine. The functional dynamics of these transporters are crucial for maintaining glucose homeostasis, and dysregulation can lead to hyperglycemia (Armour et al., 2022) and diseases like diabetes, cardiovascular disorders, and Parkinson’s (Marques et al., 2018). Therefore, the relevance of SGLTs extends beyond glucose transport, as they are involved in various disease states, including diabetes mellitus and cancer.

SGLT inhibitors also show potential applications in oncology, by blocking glucose uptake in cancer cells, impairing tumor growth (Niu et al., 2022a; Dutka et al., 2022). Several inhibitors, such as empagliflozin, dapagliflozin, canagliflozin, ertugliflozin, and bexagliflozin, SGLT2 inhibitors have been approved for managing adult patients with type 2 diabetes (Padda et al., 2022) but not type 1 diabetes, and sotagliflozin is recently approved as a dual SGLT1/SGLT2 inhibitor for treating heart failure (Packer, 2023). While dual SGLT1/2 inhibitors (e.g., sotagliflozin) have demonstrated clinical potential, achieving selective inhibition of SGLT1 remains an unresolved challenge. Despite recent progress, high-resolution target structures remain limited for several SGLT isoforms (Maccari and Ottanà, 2022), leaving inhibitors’ binding mechanism and inhibition process unclear, thus preventing a detailed understanding of their structure-activity relationship (Sun et al., 2024).

Earlier structural templates came from the bacterial sodium/galactose cotransporter from *Vibrio parahaemolyticus* (vSGLT), solved by X-ray crystallography (PDB 3DH4, 2XQ2) and homology modeling (Faham et al., 2008), which predated and informed human studies. With the advent of cryo-electron microscopy (cryo-EM), atomic-resolution structures of human SGLT1 and SGLT2 are now available, transforming our understanding of substrate/inhibitor binding and conformational dynamics (Niu et al., 2022b; Lee et al., 2024; Xin et al., 2021). Complementary computational techniques such as molecular dynamics (MD) simulations, free-energy calculations, and artificial intelligence-based structure prediction (e.g., AlphaFold2) have provided atomic-level insights into transporter flexibility, substrate and inhibitor binding mechanisms, and energy landscapes.

Therefore, this review aims to critically examine the current knowledge of human SGLT (hSGLT) proteins through the lens of computational and structural biology to understand their molecular and inhibitory mechanisms, particularly SGLT1 and SGLT2. Understanding these mechanisms is important not only for advancing our knowledge of glucose homeostasis but also for developing selective inhibitors and targeted therapies for metabolic disorders such as diabetes and certain types of cancer.

## The SGLT family

The SLC5 family includes 12 sodium-coupled transporters; SGLT1-6 are the glucose/hexose co-transporters within this family. The group also consists of myoinositol (SLC5A3 and SLC5A11), iodide (SLC5A5), vitamin (SLC5A6), choline (SLC5A7) and monocarboxylate (SLC5A8 and SLC5A12) transporters (Gyimesi et al., 2020). All SGLT transporters co-transport sodium and glucose (except SGLT3, a glucose sensor) (Nevola et al., 2023) Table 1. The named six sodium-glucose transporters have been identified in different human body tissues Figure 1. SGLT2 inhibition represents one of the most consistent and clinically validated therapeutic strategies in type 2 diabetes. Its ability to reduce glucose reabsorption has made it a cornerstone of therapy, with proven benefits on both microvascular and macrovascular complications (Nevola et al., 2023). Although developed to selectively target SGLT2, these inhibitors also interact with SGLT1 and other isoforms such as SGLT3, depending on the compound (Grempler et al., 2012a), with evidence from animal models indicating that compounds such as empagliflozin (a selective sodium glucose co-transporter 2 inhibitor) improve hyperglycaemic endothelial dysfunction through interactions with both SGLT1 and SGLT2 (Khemais-Benkhiat et al., 2020). Little information is available about other SGLT isoforms; further investigation of all SGLT’s pathophysiological mechanisms to modulate their effects could have unexpected implications.

**TABLE 1:** Summarized functions of SGLT transporters.

Protein	Function
SGLT1	Absorption of glucose, galactose, urea, and water in the intestinal epithelium (Wright et al., 2003)
SGLT2	Primary mediator of glucose and sodium reabsorption in kidney proximal tubules (Vallon et al., 2011; Wright et al., 2007)
SGLT3	Glucose sensor, with no significant transport activity (Soták et al., 2017; Bianchi and Díez-Sampedro, 2010)
SGLT4	Sodium-dependent transporter of glucose, fructose, and mannose (Tazawa et al., 2005)
SGLT5	Transporter of glucose, galactose, fructose, and mannose in kidney (Grempler et al., 2012b)
SGLT6	Transporter of glucose and myo-inositol (Coady et al., 2002; Wood and Trayhurn, 2003)

### SGLT1

The first SGLT transporter, SGLT1, was identified and cloned 27 years after Bob Crane’s 1960 proposal that active glucose absorption across the intestinal brush border is driven by ${Na}^{+}$ /glucose cotransport, with the sodium gradient providing the energy for transport (Wright et al., 2011; Wright et al., 2017; Hediger et al., 1987). Following this, human SGLT1 and SGLT2 were cloned, alongside the discovery of other members within the SLC5 gene family, which includes various transporters for different substrates (Wright et al., 2017). Mutation of SGLT1 is associated with glucose-galactose malabsorption (Xu et al., 2023). SGLT1 is a low-capacity, high-affinity ${Na}^{+}$ /glucose co-transporter with 2:1 stoichiometry expressed by the SLC5A gene located on chromosome 22q13.1 position, consisting of 664 amino acids with 14 transmembrane (TM) domains (Wright et al., 2004; Sano et al., 2020). SGLT1’s main function is the absorption of glucose and galactose across the intestinal brush border membrane (Gyimesi et al., 2020). SGLT1 transports the natural sugars glucose and galactose, but not fructose or mannose (Gyimesi et al., 2020; Kamitori et al., 2022). SGLT1 has been detected in several other tissues (lungs, liver, pancreas, immune system) (Vrhovac et al., 2015), although its role is still unknown. Cryo-EM resolved human SGLT1 bound to LX2761, showing inhibitor blockade of the water pathway, with mutagenesis and uptake assays confirming key residue interactions (Han et al., 2022). Computational docking and MD further explained SGLT1-selectivity of mizagliflozin by revealing steric clashes in SGLT2 (Han et al., 2022). Although no SGLT1 inhibitors are yet approved, candidates such as LX2761, TP0438836, and mizagliflozin are in clinical trials with potential cardiovascular benefits (Azizogli et al., 2023).

### SGLT2

SGLT2, encoded by SLC5A2 located on chromosome 16p11.2 locus, is the principal renal ${Na}^{+}$ /glucose cotransporter and a major therapeutic target with extensive preclinical and clinical characterization, with enriched expression in the early proximal tubule segments (Tönjes and Kovacs, 2013). The co-transporter is a low-affinity Michaelis-Menten constant $\left(K_{m}\right)$ ≈6 mM , high-capacity glucose symporter of about 73 kDa (Gyimesi et al., 2020; Sano et al., 2020; Nevola et al., 2023). SGLT2, a 672-amino-acid protein, is localized to the apical membrane of the S1-S2 segments of the proximal convoluted tubule, where it mediates low-affinity, high-capacity ${Na}^{+}$ /glucose cotransport with ≈ 1:1 stoichiometry. In euglycemia, the kidney filters ≈ 180 g of glucose per day; SGLT2 reabsorbs 80%–90% of this load in S1–S2, with the remainder reclaimed by SGLT1 in S3 (Coady et al., 2017a; Nevola et al., 2023). Mutations in the gene encoding SGLT2 are responsible for familial renal glucosuria (van den Heuvel et al., 2002). SGLT2 expression has been detected in the pancreas, brain, liver, thyroid, and muscle, but also in prostate tumors and glioblastoma (Nakano et al., 2022; Nevola et al., 2023; Vrhovac et al., 2015). However, no detectable SGLT2 expression is recorded in the intestinal or cardiac tissue (Gyimesi et al., 2020). SGLT2 is a promising target for a new class of drugs primarily established as kidney-targeting, effective glucose-lowering agents used in diabetes mellitus (DM) patients (Wicik et al., 2022). Increasing evidence indicates that besides renal effects, SGLT2 inhibitors (also known as gliflozins) have also a systemic impact via indirectly targeting the heart (Wicik et al., 2022). Although SGLT2 inhibitors act in the kidney, natriuresis and osmotic diuresis reduce preload/afterload and improve cardio-renal outcomes; this mechanistic link explains heart failure approvals that extend beyond glycaemia (Vallon and Verma, 2021; Fathi et al., 2021). Canagliflozin, Dapagliflozin, Empagliflozin, and recently bexagliflozin are the four main FDA-approved SGLT2 inhibitors (Gourgari et al., 2017; Khan NA. et al., 2023), and Canagliflozin is the least selective, being able to act partially on SGLT1 and other tissues (Nevola et al., 2023). Recent studies combining structural and computational approaches have deepened mechanistic understanding of SGLT2. Specifically, homology modeling using AlphaFold2 predicted key inhibitor-binding residues, which were experimentally validated through site-directed mutagenesis. Mutations at residues such as S74, D201, and F98 significantly impaired inhibitor binding and glucose transport activity, confirming their critical functional role (Hiraizumi et al., 2024). These findings underscore a strong convergence between *in silico* predictions and functional assays, supporting the structural basis of SGLT2 inhibition by gliflozins.

### SGLT3

SGLT3 is encoded by the SLC5A4 gene on chromosome 22q12.3 and consists of 660 amino acid residues. Originally cloned from colon carcinoma cDNA, it is predominantly expressed in cholinergic neurons of the enteric nervous system and is found in the neuromuscular junction of skeletal muscle, small intestine, kidney, uterus, and testis (Nevola et al., 2023; Wright, 2013; Szablewski, 2017; Soták et al., 2017). SGLT3 has been linked to the regulation of intestinal motility in response to glucose (Nevola et al., 2023). Unlike other sodium and glucose transporters, SGLT3 functions as a glucose sensor rather than a transporter (Soták et al., 2017; Delaere et al., 2013; Diez-Sampedro et al., 2003). In silico analyses identified a glutamate at position 457 as the key reason SGLT3 fails to transport glucose, a prediction confirmed experimentally: binding of extracellular glucose to hSGLT3 elicits a ${Na}^{+}$ -dependent, pH-sensitive inward current that depolarizes the membrane (glucose-gated conductance), and mutating Glu-457 restores transport, supporting its role as a glucose sensor rather than a transporter (Soták et al., 2017; Barcelona et al., 2012). SGLT3 modulation could have implications for obesity (Soták et al., 2021), metabolic syndrome, and attention-deficit hyperactivity disorder (ADHD) (Nevola et al., 2023). However, no drugs acting on SGLT3 are commercially available to date.

### SGLT4

SGLT4 (SLC5A9), located on chromosome 1p33, encodes a 699-aa sodium-dependent sugar transporter cloned from human small-intestine cDNA libraries (Szablewski, 2017; Tazawa et al., 2005). SGLT4 is expressed in the kidney, liver, lung, brain, small intestines, heart, uterus, and colon-rectal tumor (Wright, 2013; Szablewski, 2017; Gyimesi et al., 2020). SGLT4 co-transports sugars with sodium, with substrate preferences that include fructose and mannose. SGLT4’s ability to cotransport mannose and fructose with sodium arises from a more permissive, less aromatic sugar-binding cavity created by substitutions of histidine to leucine at position 83, threonine to alanine at position 287, and tyrosine to cysteine at position 290, which accommodates mannose and β -fructopyranose while preserving the sodium-coupling network (Nevola et al., 2023; Tazawa et al., 2005; Kamitori et al., 2022). It also contributes to renal mannose reabsorption and systemic mannose homeostasis (Tazawa et al., 2005). *In vivo* studies using a mouse model demonstrated SGLT4’s fructose transport capabilities compared to other SGLT isoforms (Kamitori et al., 2022). Generally, little information about SGLT4 expression and its transport physiological activity is available. SGLT4 has a potential role in the pathogenesis of diabetic proliferative retinopathy (Sánchez-Muñoz et al., 2024; Ung et al., 2017). Therapeutic implications of its modulation are not currently being evaluated.

### SGLT5

SGLT5 (SLC5A10), predominantly expressed in the renal cortex, transports mannose, fructose, and glucose. In humans, it’s located on chromosome 17p11.2 (Wright, 2013; Grempler et al., 2012b). Its primary role is the reabsorption of filtered fructose, glucose, and mannose (Gyimesi et al., 2020; Nevola et al., 2023; Mathew et al., 2024). SGLT5 has a high capacity and affinity for fructose and mannose and less affinity for galactose, glucose, and 1,5-AG (1,5-anhydroglucitol) (Grempler et al., 2012b). Recent studies reported that SGLT5 is the main 1,5-AG transporter in the kidney (Diederich et al., 2023). Preclinical studies suggest that SGLT5 may be protective in G6PC3-deficient neutropenia, likely by lowering 1,5-anhydroglucitol, though the evidence remains preliminary (Nevola et al., 2023; Diederich et al., 2023). *In vivo* mouse studies have led to the hypothesis that inhibiting SGLT5 may increase urinary fructose excretion and modulate fructose-induced hepatic steatosis (Nevola et al., 2023; Fukuzawa et al., 2013). As of now, there are no specific or safe approved SGLT5 inhibitors.

### SGLT6

SGLT6, also known as sodium/myo-inositol transporter 2 (SMIT2), is encoded by the SLC5A11 gene located on chromosome 16p12.1 (Uveges et al., 2011; Wright, 2013; Nevola et al., 2023). It functions primarily as a ${Na}^{+}$ /myo-inositol cotransporter, with additional activity toward chiro-inositol and D-glucose, and is localized to the luminal membrane of proximal convoluted tubule cells, as well as in the brain and small intestine (Uveges et al., 2011; Baader-Pagler et al., 2018). SLC5A11 gene mutations have been associated with congenital neutropenia and renal glucosuria (Youssef et al., 2023). SGLT6 inhibitors have been studied for their significance in managing diabetes conditions by lowering blood glucose levels and body weight management by increasing the urinary excretion of myo-inositol and glucose (Wood and Trayhurn, 2003). A potent, central nervous system-penetrant inhibitor ‘Cpd B’ has been developed as a selective blocker of SGLT6 (Baader-Pagler et al., 2018). There are no currently approved inhibitors specifically for SGLT6.

## SGLT structures

In this section, we review cryo-EM structures of hSGLT1/2 alongside comparative modeling and MD to interpret conformational mechanisms. Cryo-EM provides the experimental anchor, AlphaFold2 fills unresolved regions, and MD simulations test conformational dynamics. Comparisons with bacterial vSGLT highlight evolutionary conservation, while validation through mutagenesis, uptake, and binding assays creates a convergence pipeline linking structure to function.

**FIGURE 2:**
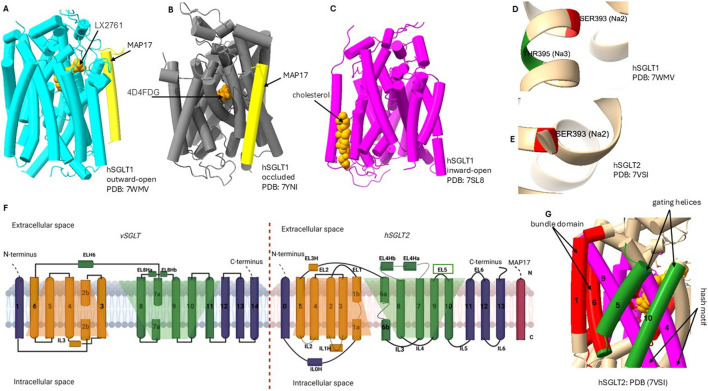
Conformational states and LeuT-fold of SGLTs. **(A–C)** hSGLT1 cryo-EM structures in outward-open (PDB 7WMV), occluded (7YNI), and inward-open (7SL8) states with bound inhibitors and MAP17. **(D–E)** the ${Na}^{+}$ sites: Na2 in both isoforms and Na3 in hSGLT1 (not resolved in hSGLT2). All 14 transmembrane helices are numbered; extracellular and intracellular sides are labeled. Shaded trapeziums (orange and green) mark the two inverted repeats of the LeuT-like fold: the bundle domain (TM1 and TM6: red), gating helice (TM5 and TM10; green) move over the hash motif (TM4 and TM9; magenta) to produce alternating access. Insets highlight the outer vestibule/lid (EL5c) in hSGLT2. Together, panels **(A–G)** provide a side-by-side comparison of states, fold elements, ligand/MAP17 positioning, and sodium-site differences relevant to isoform selectivity.

SGLTs are integral membrane proteins composed of 14 transmembrane helices arranged in a LeuT-like fold Figure 2, a hallmark of the SLC5 family. This LeuT-fold architecture underpins an alternating-access mechanism cycling between outward-open, occluded, and inward-open states to couple ${Na}^{+}$ and glucose transport. High-resolution cryo-EM studies of human SGLT1 and SGLT2 have captured these states Table 2 and defined inhibitor binding poses (Adelman et al., 2016; Cui et al., 2023). SGLT1 and SGLT2 are characterized by their structural similarities and conserved binding motifs. Structural analysis of vSGLT from *Vibrio parahaemolyticus* (PDB: 3DH4, 2XQ2) revealed a characteristic LeuT-like fold formed by the N- and C-terminal regions, although these early bacterial crystal structures failed to capture physiologically relevant inhibitor binding, limiting their pharmacological relevance (Faham et al., 2008; Han et al., 2022).

**TABLE 2:** Summary of SGLT transporter structural data: source organism, PDB and year, method of structure determination, resolution, conformation and references.

Protein	PDB ID (Year)	Organism	Method	Resolution (Å)	Conformation
vSGLT	2XQ2 (2010)	*V. parahaemolyticus*	X-ray	2.73	Inward open
	3DH4 (2008)	*V. parahaemolyticus*	X-ray	2.70	Inward open
SGLT1	7WMV (2022)	*Homo sapiens*	Cryo-EM	3.20	Outward open
	7YNI (2023)	*Homo sapiens*	Cryo-EM	3.26	Occluded
	7SLA (2021)	*Homo sapiens*	Cryo-EM	3.15	Inward open
	7SL8 (2021)	*Homo sapiens*	Cryo-EM	3.40	Inward open
SGLT2	7VSI (2022)	*Homo sapiens*	Cryo-EM	2.95	Outward open
	7YNJ (2023)	*Homo sapiens*	Cryo-EM	3.33	Occluded
	8HIN (2023)	*Homo sapiens*	Cryo-EM	3.30	Inward open
	8HEZ (2023)	*Homo sapiens*	Cryo-EM	2.80	Outward open
	8HG7 (2023)	*Homo sapiens*	Cryo-EM	3.10	Outward open

Recent advancements in SGLT studies with Cryo-EM have provided significant insights into the three-dimensional structural organization and binding modes of inhibitors of human SGLT1 and SGLT2 (Niu et al., 2022b; Han et al., 2022). Currently, only human SGLT1 and SGLT2 structures are experimentally resolved. By contrast, SGLT3, SGLT4, SGLT5, and SGLT6 lack experimental structures and are investigated through homology modeling. Studies showed that, although hSGLT1 possesses an extracellular “lid” domain absent in vSGLT Figure 2, its overall architecture is conserved: 14 transmembrane helices (TM0–TM13) arranged in an amino acid-polyamine-organocation (APC) fold with two inverted repeats (TM1-5 and TM6-10) (Han et al., 2022; Krofchick, 2012). Similarly, hSGLT2 has 14 transmembrane helices arranged in two structurally similar but topologically inverted repeats (Niu et al., 2022a). Cryo-EM further demonstrated the conservation of these features with bacterial vSGLT, supporting a shared transport mechanism (Niu et al., 2022a; Han et al., 2022). This mechanism allows transporter transitions from outward-open conformation to inward-open conformation, facilitating the binding and release of sodium and glucose (Adelman et al., 2016). Notably, SGLT transport is symport: both ${Na}^{+}$ and glucose are carried into the cell together (Niu et al., 2022a). SGLT conformational structural changes involve flexible “moving” helices (hash domain: TM3, TM4, TM8, TM9) and more stable “bundle domain” helices (TM1, TM2, TM6, TM7), with TM5 and TM10 forming gating helices Figure 2 (Cui et al., 2023).

Molecular dynamics (MD) simulations have provided important insights into the dynamics of substrate binding and translocation in SGLTs. In hSGLT1 structure, MD confirmed the binding sites for cholesterol, glucose, and ${Na}^{+}$ , and MD simulations were critical for visualizing outward-open to inward-open transitions and probing ${Na}^{+}$ binding stability at the Na2/Na3 sites (here, Na2 is a conserved sodium-binding site found in all SGLTs, while Na3 is an additional site unique to SGLT1) Figure 2, offering dynamic insights beyond static cryo-EM structures (Han et al., 2022; Niu et al., 2022b). Together with experimental evidence, these computational studies identified specific amino acid residues involved in substrate binding and transport, underscoring the conservation of key residues across the SGLT family (Niu et al., 2022a; Cui et al., 2023; Han et al., 2022; Ghezzi et al., 2014; Hiraizumi et al., 2024).

**FIGURE 3:**
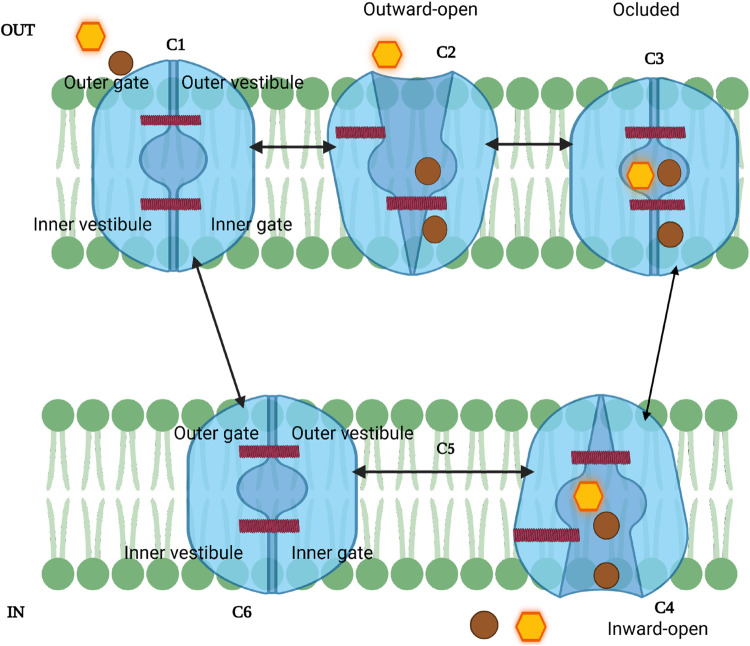
Schematic model of the SGLT sodium-glucose transport cycle. The transporter alternates between outward-facing (C1-C2), occluded (C3), and inward-facing (C4-C6) conformations to couple Na^+^ (brown circles) and glucose (yellow hexagon) translocation. Sodium binds first, followed by glucose, triggering conformational changes that open the inner gate and release substrates into the cytoplasm. C5 represents a transient intermediate; because sodium release can occur stochastically, this step may be bypassed. The cycle resets rapidly (C6→C1) via the “rocking bundle” mechanism.

Sodium ions play a significant role in the functioning of sodium-glucose cotransporters SGLT1 and SGLT2, which are critical for glucose absorption and homeostasis. Since sodium is transported along with its concentration gradient, it serves as a source of energy crucial for SGLT to transport glucose against its concentration gradient into the cells (Abdul-Ghani et al., 2011). ${Na}^{+}$ was reported to be the most effective ion for SGLT1 activities (Poulsen et al., 2015). Sodium binds to the cotransporters, triggering a conformational change that exposes the sugar binding site Figure 3 (Poulsen et al., 2015). Sodium glucose transporters have conserved sodium-binding sites Na2 site in both SGLT1/2 and Na3 present in SGLT1 only Figure 2; these sites are important for the symport mechanisms. Binding of ${Na}^{+}$ to transporters stabilizes the outward-facing conformation, and its release facilitates a shift to the inward-open state (Hiraizumi et al., 2024; Tavoulari et al., 2016). The studies of the roles of ${Na}^{+}$ in vSGLT show that the binding of ${Na}^{+}$ triggers a conformational change to outward-facing, and without ${Na}^{+}$ , this conformation is less likely to occur (Hiraizumi et al., 2024). These findings prove that sodium binding is the critical step in this process, leading to structural rearrangements that prime the transporter for substrate interaction.

The SGLT transport cycle starts in an outward-open state (C1–C2), exposed to extracellular ${Na}^{+}$ Figure 3. ${Na}^{+}$ first binds at Na2 (and, in hSGLT1, also at Na3), stabilizing the outward-open conformation and priming glucose binding, after which gating proceeds to the occluded state (state 2) (Bisignano et al., 2020; Wright et al., 2017; Cui et al., 2023). This conformational change is important for the subsequent binding of glucose or galactose (vSGLT), which occurs in state 3 (occluded conformation), causing the closure of the external gate (C2-C3) (Khan F. et al., 2023; Wright et al., 2017). The sugar substrates are found to be confined within the cavity and surrounded by TM1, TM2, TM3, TM7, and TM10 (Cui et al., 2023). The binding of glucose and sodium triggers a significant isomerization of the transporter, resulting in a shift to an inward-facing conformation (C4-C6) or state 4; in this state, the internal gate opens, releasing sodium and glucose into the cytoplasm (Wright et al., 2017; Cui et al., 2023). However, studies found that the release of ${Na}^{+}$ is stochastic and not strictly ordered with sugar release; that means the ${Na}^{+}$ can exit the transporter before sugar is released into the inner compartment (Adelman et al., 2016). Since the intracellular release of the ligand is considered stochastic, the transition from the inward-open conformation can be viewed without C5 state (Wright, 2021). The final step is marked by transitioning from inward open to outward open conformation (C6-C1) (Cui et al., 2023); during this step, the studies of vSGLT suggested that resetting the cycle utilizes a set of gating charge residues resulting in stabilizing its outward-facing conformation (Khan F. et al., 2023). These transitions are completed within ∼20 ms (Wright et al., 2017). To further explain the stochastic steps involved in glucose transport and the contribution of inhibitors on this process, SGLT2 studies suggested a new seven-state mechanism model which demonstrates how ${Na}^{+}$ ion can exit before the sugar is released into the proximal tubule epithelial cells, and the binding of ${Na}^{+}$ and glucose in inward-facing conformation occurs in an unordered manner (Barreto and Alencar, 2022). Therefore, SGLTs function via a “rocking bundle” principle, with conformational changes throughout sugar transport. Understanding how different inhibitors interact with SGLTs at specific state conformational states may inform the design of more effective and selective therapeutics for diabetes and cancer.

SGLT transporters assemble as dimers, where two protomers form a functional unit in the membrane. Structural and biophysical studies show that dimerization stabilizes the transporter and supports proper trafficking. Accordingly, the oligomeric state should be considered when interpreting SGLT function and when designing SGLT1-selective inhibitors later in this review. The concept of dimerization among SGLTs, particularly SGLT1, is gaining attention among scientific research. SGLT1 dimeric forms demonstrated experimentally by Förster resonance energy transfer (FRET) signals, which imply physical interactions between SGLT1 monomers within cellular membranes (Sarabipour and Hristova, 2013). Other studies using the same experimental technique revealed that hSGLT1 forms a disulfide-bridged homodimer, with C355 being crucial for this association, and suggest that this dimerization impacts its functional properties (Sasseville et al., 2016). These biochemical features are pivotal, as the transition between monomeric and dimeric states may influence transporter kinetics and the functional outcome of glucose uptake. The studies of vibrio sodium/galactose transporter (vSGLT), a SGLTs model using distance measurements for Electron Paramagnetic Resonance spectroscopy (DEER) revealed that the transporter can form stable dimers in solution (Paz et al., 2018). This stability indicated that the dimers do not undergo significant rearrangement during the transport cycle, making them potential targets for novel inhibitors (Paz et al., 2018).

Recent studies have highlighted the formation of hetero-dimer complexes involving sodium and glucose transporters (SGLT) with MAP17 protein. The study of human SGLT1 (hSGLT1) interaction with MAP17 forms a hetero-dimeric complex, which stabilizes its outward-open conformation, crucial for the glucose transport mechanism (Niu et al., 2022b). MAP17 has been proposed as a therapeutic target in cancer because it enhances SGLT1 activity, increasing glucose uptake and supporting tumor growth. Co-expression of MAP17 and SGLT1 has also been reported as a biomarker associated with tumor expression and, in some cohorts, favorable prognosis (Perez et al., 2013; Du et al., 2022; Tampakis et al., 2021). In SGLT2, the interaction with MAP17 was reported to increase the transporter activity and surface expression; the study demonstrated the structural mechanisms of glucose transport and inhibition, highlighting the role of ${Na}^{+}$ in stabilizing the outward-facing conformation (Hiraizumi et al., 2024; Niu et al., 2022a; Coady et al., 2017b). The role of MAP17 in hSGLT2/hSGLT1 is strongly supported by computational predictions, resolved cryo-EM heterodimers, and functional assays demonstrating MAP17-dependent expression and transport (Niu et al., 2022c; Niu et al., 2022a; Hiraizumi et al., 2024).

To add more insights into protein structure prediction advancement, in this review we used AlphaFold2 predicted SGLTs structures to assess their accuracy compared with experimental structures from Cryo-EM and X-ray crystallography. AlphaFold is an artificial intelligence (AI) system which recently gained much attention due to its proposed ability as a computational approach to accurately predict 3D protein structure even in the absence of known similar structures (Jumper et al., 2021). We used VMD 1.9.4 Multiseq tool to make a comparative analysis of vSGLT (PDB: 2XQ2), SGLTI (PDB:7WMV), SGLT2 (PDB:7VSI) structures with AlphaFold predicted structures of SGLT1 (Uniprot: P13866) and SGLT2 (Uniprot: P31639). Structures identities were assessed based on QH value, root mean square deviation (RMSD) and percentage identity score. Q is the metric for structure conservation (Eastwood et al., 2001). Q = 1 represents perfect structural match, while Q = (0.1–0.3) denotes weak alignment with minimal atom overlap (Hsin et al., 2008).

**FIGURE 4:**
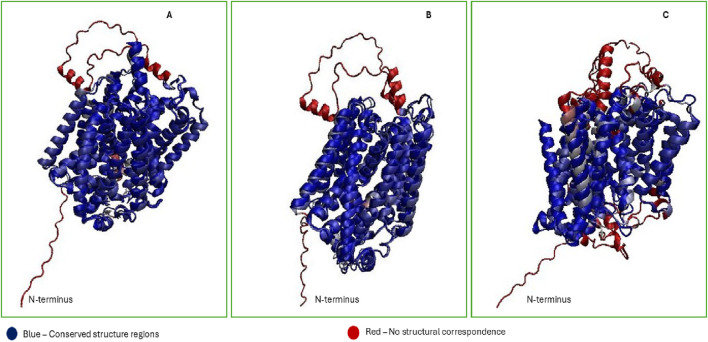
Structural comparison of experimental and AlphaFold2-predicted SGLT models **(A–C)**. **(A)** Cryo-EM structures of human SGLT1 (PDB: 7WMV), **(B)** hSGLT2 (PDB: 7VSI), and **(C)** the X-ray structure of bacterial vSGLT (PDB: 2XQ2), were aligned with AlphaFold-predicted models (UniProt: P13866 and P31639) using VMD Multiseq. RMSD and QH metrics reveal high structural agreement for SGLT1 (QH = 0.75, RMSD = 1.38 Å) and SGLT2 (QH = 0.72, RMSD = 1.17 Å), validating AlphaFold’s predictive accuracy. In contrast, vSGLT showed weak alignment (QH = 0.49, RMSD = 2.64 Å), reflecting evolutionary divergence. Residual differences, particularly in N-terminal coils and loop regions, highlight the value of cryo-EM for resolving dynamic domains in membrane transporters.

Figure 4 shows that cryo-EM-derived structures of SGLT1 and SGLT2 align closely with their AlphaFold2-predicted models, yielding high structural agreement scores (QH = 0.75, RMSD = 1.38 Åfor SGLT1; QH = 0.72, RMSD = 1.17 Åfor SGLT2). On the other hand, the results of the vSGLT structure (X-ray crystallography method) aligned with the SGLT1 AlphaFold2 predicted structure indicated QH = 0.49, RMSD = 2.64 and percentage identity = 18.02%; this implies that the structural alignment is low in structural homology compared to SGLT1 and SGLT2. The weaker alignment of vSGLT with human isoforms reflects evolutionary divergence and methodological differences; cryo-EM is particularly advantageous for membrane proteins because it preserves native-like states (Benjin and Ling, 2020).

However, the N-terminal region of all SGLT (residues 1–20) was predicted by AlphaFold2 to form a coil with low structural confidence Figure 4, and it is possible that this region remains unstructured in isolation and may only adopt a defined conformation upon membrane association or partner interaction. This observation aligns with prior findings that AlphaFold2’s predictive accuracy decreases in flexible loops exceeding 20 residues, likely due to increased lack of stable secondary structure (Stevens and He, 2022). These results underline the significance of cryo-EM-derived structures for studying hSGLTs, as they provide much closer similarities to the native human transporter compared to the homologous structure of vSGLT. As summarized in Table 2, most high-resolution human transporter protein (SGLT1/2) structures were obtained relatively recently (2021–2023), reflecting advances in cryo-EM and growing interest in these transporters as therapeutic targets.

## SGLT inhibitors

Emerging evidence indicates that SGLT oligomerization/dimerization shapes transporter function and extracellular vestibule geometry; in parallel, hSGLT2 requires the accessory partner MAP17 for robust expression and activity (Cui et al., 2023). Accordingly, inhibitor design and interpretation should consider the oligomeric state, especially for efforts aiming at SGLT1 selectivity, where dimerization may modulate gating and ligand access.

SGLT inhibitors lower glucose by blocking renal reabsorption; this concept was first demonstrated with a natural inhibitor, phlorizin, discovered from the bark of the apple tree in 1835 (Helvacı and Helvacı, 2023). Phlorizin, a natural O-glycosidic product, was identified as an early inhibitor of sodium-glucose cotransporters (SGLTs), but its clinical use was precluded due to pharmacokinetic and safety issues (poor oral bioavailability and gastrointestinal side effects) (Da Silva et al., 2018). Consequently, the focus shifted to developing new drugs based on phlorizin’s structure but optimized for SGLT-2 selectivity and improved pharmacokinetic profiles. Sodium-glucose cotransporter inhibitors were initially approved as a therapy for lowering blood sugar in adults with type 2 diabetes, but later demonstrated benefits in cardiovascular, respiratory, cancer, dementia, and chronic kidney disease, and improved overall life expectancy (O’Keefe et al., 2023). Most FDA-approved sodium-glucose cotransporter-2 (SGLT2) inhibitors are selective for SGLT2 over SGLT1. Since the discovery of the SGLT family, sustained medicinal chemistry efforts have produced multiple gliflozins with favorable efficacy and safety profiles. Regulatory agencies worldwide (e.g., FDA, EMA) have approved several prescription SGLT2-selective agents for type 2 diabetes and cardio-renal indications Table 3. Although selectivity varies by the core chemical structure of the inhibitor (scaffold), the approved class generally favors SGLT2; by contrast, dual-acting compounds (e.g., sotagliflozin) retain measurable SGLT1 inhibition (Haas et al., 2014; Packer, 2023; Šaler et al., 2024; Dominguez Rieg and Rieg, 2019). In silico virtual screening and docking against hSGLT2 (7VSI) and hSGLT1 (7WMV) yielded 20 leads, and glucose-uptake assays in SGLT2-expressing HEK293T cells confirmed 13 non-toxic inhibitors (Liu et al., 2025). Docking predicted conserved H-bonds to His80, Lys154, and Tyr290 that persisted in short MD simulations ( ≈ 5 ns per complex), whereas analogous networks were absent in hSGLT1, supporting SGLT2 selectivity (Liu et al., 2025). Together, the experimental validation and molecular docking validate the computational predictions.

**TABLE 3:** Inhibitor landscape across SGLT isoforms: studied agents, selectivity, regulatory status, and (where applicable) dose/indications (Dominguez Rieg and Rieg, 2019).

Isoform	Representative inhibitor(s)	SGLT2/1 selectivity (fold)	Year	Dose (mg/day)	Regulatory status	Indications/Notes
SGLT2	Canagliflozin	∼ 260	2013	100–300	Approved (US/EU/JP)	T2DM; T2DM + CV disease
	Dapagliflozin	∼ 1,200	2014	5–10	Approved (US/EU/JP)	T2DM; T2DM + CV disease; T2DM + HF; HF
	Empagliflozin	∼ 2,700	2014	10–25	Approved (US/EU/JP)	T2DM; T2DM + CV disease
	Ertugliflozin	∼ 2,200	2017	5–15	Approved (US)	T2DM
	Bexagliflozin	∼ 2,400	2023	20	Approved (US)	T2DM
	Ipragliflozin	∼ 250	2014	50	Approved (JP only)	Regional approval
	Luseogliflozin	∼ 1770	2014	5	Approved (JP only)	Regional approval
	Tofogliflozin	∼ 2,900	2014	20	Approved (JP only)	Regional approval
Dual SGLT1/2	Sotagliflozin	∼ 20	2023	200–400	Approved (selected regions)	HF with/without T2DM
	LX–2761	∼ 0.8	–	–	Not approved (investigational)	Gut-restricted SGLT1 design
SGLT1	Mizagliflozin	∼ 303	–	–	Not approved (investigational)	Clinical development
	KGA–2727	∼ 140	–	–	Not approved (investigational)	Clinical development
SGLT3	None reported (selective inhibitor)	–	–	–	Not approved	Glucose sensor ( ${Na}^{+}$ -gated conductance), not a transporter
SGLT4	None reported (selective inhibitor)	–	–	–	Not approved	Mannose/fructose cotransporter; no clinical agents
SGLT5	None reported (clinical inhibitor)	–	–	–	Not approved	Renal 1,5-anhydroglucitol/hexose handling; emerging target
SGLT6	Cpd B (tool compound)	–	–	–	Not approved (tool only)	CNS-penetrant selective SGLT6 blocker (research)

Most marketed agents are SGLT2-selective gliflozins. No inhibitor is approved for SGLT3–SGLT6. “JP, only” indicates approval in Japan but not in the US/EU., Doses apply only to approved products and reflect label ranges. Abbreviations: US, United state; EU, Eeuropean union; JP, Japan; T2DM, type 2 diabetes mellitus; HF, heart failure; CV, cardiovascular.

Sodium glucose transporter 2 (SGLT2) works by inhibiting the reabsorption of glucose in the kidneys, which results in increased glucose excretion in the urine, hence lowering blood glucose levels. The therapeutic benefits of these inhibitors are triggered by their unique mechanism of action, which is insulin independent (Çakmak, 2024). Several SGLT2 inhibitors, including canagliflozin, dapagliflozin, empagliflozin, ertugliflozin, and bexagliflozin have been approved by the FDA for type 2 diabetes since 2013 (Basak et al., 2023). In Japan, all three agents ipragliflozin, luseogliflozin, and tofogliflozin are approved SGLT2 inhibitors (Kshirsagar et al., 2020); in the United States and European Union, they are not approved (some have been evaluated in regional clinical programs) (Haas et al., 2014; Basak et al., 2023). To date, there are few reports of SGLT1-specific inhibitors, and the effort has not yet gained Food and Drug Administration approval for the treatment of diabetes (Wright, 2021). The development of SGLT1/SGLT2 dual inhibitors has received substantial attention from pharmaceutical companies due to their better glycaemic control and fewer side effects (Xin et al., 2021; Dominguez Rieg and Rieg, 2019). Therefore, dual inhibitors of SGLT1 and SGLT2 (such as sotagliflozin and LX-2761) are being developed for the treatment of diabetes (Powell et al., 2017; Sands et al., 2015). Sotagliflozin was recently approved by the FDA as a dual SGLT1/SGLT2 inhibitor to reduce the risk of cardiovascular death, hospitalization for heart failure, and urgent heart failure visits in adults with heart failure or type 2 diabetes, chronic kidney disease, and other cardiovascular risk factors (Packer, 2023). Safety trade-offs with current SGLT2 inhibitors (infections, diabetic ketoacidosis risk, hypotension) argue for isoform-selective or gut/kidney-targeted SGLT agents that preserve efficacy with fewer side effects (Padda et al., 2022).

## Binding mechanism of SGLT inhibitors

The binding modes of sodium-glucose cotransporter (SGLT) inhibitors are important for understanding their therapeutic potential. Structural insights into these transporters, particularly through cryo-electron microscopy (cryo-EM), have elucidated the differences in binding conformations that occur when various inhibitors interact with SGLTs (Hiraizumi et al., 2024). Inhibitors, both natural (e.g., phlorizin) and synthetic (e.g., gliflozins), typically bind with the sugar moiety in the sugar cavity and aglycon moiety in the extracellular vestibule (Bisignano et al., 2018). Binding stabilizes the outward-facing conformation, preventing the transition to an inward-open state. This blocks glucose reabsorption, which is therapeutic for conditions like type 2 diabetes (Hiraizumi et al., 2024; Han et al., 2022).

The studies found that all gliflozin inhibitors, canagliflozin, dapagliflozin, sotagliflozin, TA-1887, and empagliflozin bind to the outward-facing conformation of SGLT2 in the central hydrophobic cavity formed by transmembrane domains 1, 2, 3, 6, and 10 (Hiraizumi et al., 2024). These inhibitors utilize a glucose moiety that interacts via hydrogen bonds with residues such as N75, H80, E99, S287, W291, K321, and Q457, along with hydrophobic interactions from residues F98, L84, V98, and F453 contributed by the aglycone moiety; F98 and F453 are important for inhibitor binding in both outward-facing and inward-open conformations (Hiraizumi et al., 2024). The study of LX-2761 dual inhibitor interaction with SGLT1 reported the formation of hydrogen bonding interactions with N78, H83, E102, K321, Q457, W291, and T287 at the glucose ring, hydrophobic interactions with residues I98, F101, L274, F453, T460, L286, and M283 observed at the aglycone group, and D454 residue identified as the critical residue as its mutation decreases the potency of LX-2761 (Niu et al., 2022a). The observation of common residues by different authors implies its conservation across SGLTs, which may suggest that inhibitors’ binding modes are also conserved across transporters (Hiraizumi et al., 2024; Bisignano et al., 2018; Han et al., 2022; Wright, 2021). The natural phlorizin inhibitor was found to bind to TM1, TM5, and TM8 in an inward–open structure; the Cryo-EM structure captured phlorizin bound to the intracellular sides in the inward-open conformation (Hiraizumi et al., 2024). Researchers found that phlorizin exhibits biphasic binding kinetics, suggesting that it can bind to both extracellular and intracellular sides of SGLT2; however, the study suggests that the intracellular binding site has lower affinity, meaning that it requires a high concentration of phlorizin to be effective from inside (Hiraizumi et al., 2024). This indicates that phlorizin might have a more complex mechanism of action than gliflozins, potentially interfering with both glucose uptake and release. The binding site for phlorizin was found to be located near the Na2 site (Hiraizumi et al., 2024; Wright, 2021), but the exact nature of this interaction and conformations remains unresolved. Consistent with this, MD simulations of structural models align with cryo-EM in confirming outward-open ${Na}^{+}$ -bound and inward-open ${Na}^{+}$ -free states, yet several questions persist,the Na3 site is occupied and functional in SGLT1 but ${Na}^{+}$ density is absent at Na3 in SGLT2, and while hSGLT2 requires MAP17 (PDZK1IP1) an accessory membrane protein that activates and stabilizes hSGLT2 to increase its functional activity, hSGLT1 can function without it despite evidence of MAP17 interaction (Niu et al., 2022b).

Computational and functional studies of SGLT1 and SGLT2 with phlorizin and dapagliflozin showed binding in the outward-open conformation; however, key differences emerged in the C-terminal segment extracellular loop (EL5c) and in the presence of an additional Na3 binding site in SGLT1, which is absent in SGLT2 and vSGLT (Bisignano et al., 2018). The study observed the structural differences between SGLT2 and SGLT1 at the EL5c loop which contribute to inhibition selectivity. SGLT2 was found to have a histidine (H268) aromatic residue at this position of the loop, which creates an aromatic cage (H80, F98, and H268) around the central ring of the aglycone tail and thus interacts more strongly with inhibitors, while SGLT1 does not form an aromatic cage because it has aspartic acid at this loop and interacts less strongly with inhibitors (Bisignano et al., 2018). Studies on dual inhibitors (sotagliflozin, LX-2761) revealed structural pocket differences between SGLT1 and SGLT2 that explain selectivity (Hiraizumi et al., 2024); however, dynamic insights remain speculative, as the role of V157 in SGLT2 (corresponding to A160 in SGLT1) was suggested by MD simulation but not directly tested.

The reported key fundamental kinetic differences between SGLT1 and SGLT2 are stoichiometry sodium ion glucose coupling ratio, which are 2:1 for SGLT1 while 1:1 for SGLT2; the ability of SGLT1 to transport galactose while SGLT2 shows a reduced ability for galactose transport; high selectivity of SGLT2 for inhibitors compared to SGLT1; the need of MAP17 by SGLT2 to facilitate its surface expression; and the capacitive current demonstrated by SGLT1, which is absent in SGLT2 (Han et al., 2022; Wright, 2021; Cui et al., 2023; Hiraizumi et al., 2024).

To gain more insights on the degree of binding interaction between inhibitors with SGLT1 and SGLT2 based on binding free energy ΔG , many researchers have utilized Molecular Mechanics, General Born Surface Area (MM/GBSA) (Hayes and Archontis, 2012; Xin et al., 2021). The binding energy of SGLT1 and SGLT2 inhibitors is important for determining the efficacy of therapeutic agents by measuring how strongly a compound binds to the SGLT protein, and ultimately designing novel SGLT inhibitors for conditions like diabetes. Empagliflozin, ertugliflozin, and LX2761 show more favorable (more negative) binding free energies than glucose Table 4, indicating thermodynamically stronger binding to the transporters (Pang et al., 2023).

**TABLE 4:** Binding energies of different SGLT inhibitors and natural substrates calculated using the MM/GBSA method.

Ligand	Target	Binding energy (kcal/mol)	Source
Ertugliflozin	SGLT1	−82.23	Xin et al. (2021)
	SGLT2	−41.45	
Empagliflozin	SGLT2	−68.10	
Sotagliflozin	SGLT1	−74.58	
	SGLT2	−70.83	
LX2761	SGLT1	−86.67	Pang et al. (2023)
	SGLT2	−63.42	
α -glucose	SGLT1	−8.89	
	SGLT2	−24.70	
β -glucose	SGLT1	−16.00	
	SGLT2	−14.20	

Across SGLT1 and SGLT2, MM/GBSA rescoring reproduced the experimental potency order; gliflozins showed more favorable (more negative) binding free energies than glucose and identified residue-level determinants (example, Y290/F101 in hSGLT1; H80/F98/H268 in hSGLT2/EL5c) that were subsequently validated by mutagenesis and electrophysiology (Bisignano et al., 2018). In hSGLT1, predicted sugar-pocket and aglycone-vestibule contacts (example, Y290 in the sugar pocket; F101 π – π stacking to the aglycone) were supported by site-directed mutagenesis and α -methyl-D-glucose ( α -MG) uptake/binding assays, which selectively weakened inhibitor potency while preserving glucose transport (Bisignano et al., 2018). In hSGLT2, the models predicted that a third aromatic residue, H268, stacks with F98 to form an ‘aromatic cage’ around the central ring of the aglycone tail, enhancing packing interactions; the feature was absent in hSGLT1. This structural insight from the models was directly supported by experimental mutagenesis studies: mutating hSGLT1 D268 to H (mimicking hSGLT2’s EL5c) led to a 13-fold increase in dapagliflozin potency for hSGLT1 (Bisignano et al., 2018). Thus, MM/GBSA serves as a reliable pose-refinement/ranking method whose contact maps align with structure-function data and guide cryo-EM/MD follow-up.

## Water permeation

Beyond glucose transport, the SGLT family, especially SGLT1, demonstrates a dual role where they facilitate water permeation through a channel-like pathway (Han et al., 2022). Water transport follows similar pathways as ${Na}^{+}$ and glucose, and its permeation is independent of the presence of ${Na}^{+}$ and glucose (Wright et al., 2017). Water transport across the cell membrane is important for cellular homeostasis (Adelman et al., 2014). Two main hypotheses have been used to study the water transport mechanism across SGLT transporters, particularly SGLT1,which are passive or osmotic-dependent transport and active or cotransport-dependent transport hypotheses (Loo et al., 2002). The studies revealed that passive transport requires an open conformation of the transporter, while active transport involves conformational changes (Sever and Merzel, 2023a). The studies of human SGLT1 suggest that the water-permeation pathway depends on the conformational changes in the transporter, and the movement of transmembrane segments TM10 and TM1 Figure 2F creates a narrow opening that connects to the extracellular solution, facilitating passive water transport (Han et al., 2022). The computational studies of the effects of different inhibitors (phlorizin, mizagliflozin, and sotagliflozin) in the water transport mechanism across SGLT1 channels at different states show that the system with bound inhibitors significantly reduces water permeability, while the apo and galactose-bound state increases water permeability (Sever and Merzel, 2023a). The same study identified water permeating pathways which seem similar in both extracellular and intracellular directions, suggesting a passive mechanism, and hash motif and bundle domain residues of SGLT1 were critical for water transport (Sever and Merzel, 2023a). The lining pore residues F453 and Q454 on TM10 are crucial for the SGLT1 water permeation (Han et al., 2022; Sever and Merzel, 2023a). Mutations in these residues can restrict water permeability, emphasizing the significance of structural integrity for this function. Using all-atom MD simulation, it was concluded that water follows passive transport which is influenced by SGLT1 intrinsic dynamic flexibility (Sever and Merzel, 2023b).

Computational MD simulations have shown that the dynamics of water molecules in the binding site can significantly affect the binding energy and overall efficacy of the inhibitors (Bisignano et al., 2018). Water molecules can mediate interactions between the inhibitor and the transporter, influencing the stability of the binding complex (García-Sosa, 2013). 3D-RISM (three-dimensional reference interaction site model) water analysis predicted stable hydration near gliflozin binding sites, explaining the higher affinity of inhibitors like dapagliflozin. This computational insight aligned with mutagenesis experiments, where mutating nearby residues reduced binding, confirming the role of water-mediated interactions in stabilizing inhibitor binding. These water and contact maps center on the EL5c/TM10 region and align with mutagenesis effects at F98 (TM2) and F453 (TM10) (Hiraizumi et al., 2024). Finally, molecular dynamics and cryo-EM of hSGLT1 structure indicate a closed water pathway in inhibitor-bound states (Niu et al., 2022b), but whether similar gating occurs in SGLT2 remains uncertain. Generally, inhibitor chemotypes that block the TM10/TM1 water pathway in SGLT1 may reduce transmembrane water permeability and should be monitored during the development of intestine-restricted SGLT1 blockers.

## Clinical implications of SGLT inhibition

Sodium-glucose cotransporter (SGLT) inhibitors represent a significant class of medication that has been shown to offer multifaceted benefits in the management of diabetes, especially type 2 diabetes mellitus; this significant clinical implication extends beyond glycaemic control. Their role includes cardiovascular protection, renal outcomes, and loss of body weight, which present novel therapeutic avenues for patients with diabetes, particularly those at risk for heart failure (HF) and chronic kidney disease (CKD). SGLTs have also been studied for their potential implications in cancer-related diseases.

Studies show that empagliflozin, dapagliflozin, and canagliflozin, SGLT-2 inhibitors, primarily function by promoting glucosuria, thereby reducing plasma glucose levels and improving glycaemic control in diabetic patients (Basak et al., 2023; Koh and Chung, 2024; Komoroski et al., 2009). The randomized trials of SGLT2 inhibitors reported improvement in cardiovascular and kidney health, reducing heart failure hospitalizations even in non-diabetic patients with reduced ejection fraction heart failure (Cowie and Fisher, 2020; Fernandez-Fernandez et al., 2020). Other recent studies found similar benefits in the inhibitors but cautioned the patients to use the drugs under controlled conditions as they are associated with an increased risk of genital mycotic infections and a small increased risk of diabetic ketoacidosis (O’Hara et al., 2024).

SGLT1 inhibitors, on the other hand, have gained significant attention in recent years due to their crucial role in glucose absorption in the intestine and their potential implications in diabetes management. The studies have demonstrated that SGLT1 inhibition can enhance glucose homeostasis by reducing intestinal glucose absorption, promoting the release of glucagon-like peptide-1, and the potential for treating its associated cardiac abnormalities (Zhao et al., 2023; Kalra et al., 2020). SGLT1 inhibitors indicated improved glycemic control by reducing glycosylated hemoglobin (HbA1c) levels by approximately 0.29%–0.4% and fasting plasma glucose by 0.85–1.32 mmol/L (Zou et al., 2021; Popovic et al., 2024). Recently, Sotagliflozin, a dual SGLT1/2 inhibitor, has gained FDA approval for reducing the risk of cardiovascular death, hospitalization for heart failure, and urgent heart failure visits in patients with heart failure or type 2 diabetes, CKD, and other cardiovascular risk factors (Packer, 2023). However, their use requires careful patient selection to mitigate risks, particularly ketoacidosis, and to minimize other reported adverse effects (Janssens et al., 2020; Edwards et al., 2023; von Scholten et al., 2021; Wright, 2020).

Beyond their antidiabetic effects, SGLT inhibitors have also shown promising benefits in various malignancies. Evidence indicates that SGLT-2 inhibitors can inhibit cancer cell proliferation, particularly in breast, pancreatic, colon, and prostate cancers. These effects are hypothesized to result from the inhibition of glucose uptake, which is essential for tumor cell viability (Dabrowski, 2021; Dutka et al., 2022; Zhou et al., 2020; Komatsu et al., 2020). For instance, studies have noted that SGLT-2 inhibition lowers blood glucose supply to tumors, thereby limiting their growth and survival (Shi et al., 2021; Scafoglio et al., 2018). The recent findings underscore the potential of SGLT-2 inhibitors, especially in combination with glucagon-like peptide-1 receptor agonists (GLP-1), to improve survival outcomes in lung cancer patients with type 2 diabetes (Chen et al., 2025). Several studies have demonstrated the efficacy of SGLT-2 inhibitors against cancers such as prostate and breast cancer (Aber et al., 2025; Karim et al., 2024; Anastasio et al., 2024; Sokołowska et al., 2025).

Research indicates that SGLT1 is a crucial factor for the survival and proliferation of certain cancer cells. For instance, in triple-negative breast cancer (TNBC) models, SGLT1 expression significantly correlated with tumor size and clinical-pathological characteristics, presenting SGLT1 as a prognostic marker (Liu et al., 2019) and its SGLT1 overexpression correlates with worse outcomes in colorectal cancer patients (Guo et al., 2011). Beyond breast and prostate cancer, the evidence shows that SGLT1 plays a role in other malignancies, including cervical cancer (Perez et al., 2013). Preclinical studies and reviews highlight that repurposing available SGLT2 or SGLT1/SGLT2 inhibitors traditionally developed for diabetes mellitus management might be a novel option for cancer therapy (Vrhovac Madunić et al., 2018; Pandey et al., 2025), as some, such as empagliflozin, demonstrated promising results for cancer treatment (Wu et al., 2023; Eliaa et al., 2020). However, this poses challenges regarding selectivity and the optimization of dosing regimens. Therefore, focusing on designing inhibitors with improved specificity could be a promising area for cancer and diabetes-related disorders.

## The unmet need for selective SGLT1 inhibition

The landscape of sodium-glucose transporter (SGLT) inhibitors has seen significant advancements, particularly with the approval of numerous SGLT2 inhibitors for managing type 2 diabetes and its cardiovascular and renal complications. However, despite these successes, there remains a critical unmet need for the development and approval of truly selective SGLT1 inhibitors. Selective SGLT1 inhibition offers distinct therapeutic advantages due to SGLT1’s unique physiological roles and expression patterns. Therefore, designing inhibitors with improved specificity for SGLT1 could be a promising area for both cancer and diabetes-related disorders.

To overcome the challenges of structural and binding similarities in human SGLT while developing selective SGLT1 inhibitors, future research should focus on exploiting unique structural features of SGLT1, such as the Na3 binding site, which is present in SGLT1 but absent in SGLT2 and vSGLT (Bisignano et al., 2018). “Design perspective: Therefore, SGLT1 selectivity can be pursued by targeting the Na3 site, leveraging EL5c-specific polar residues that can form H-bond/salt-bridge contacts with the aglycone of inhibitors, and exploiting the larger central pocket of SGLT1 while avoiding hydrophobic interactions favored in SGLT2.”

Current SGLT inhibitors primarily act by binding to the orthosteric (active) site, directly competing with glucose and/or ${Na}^{+}$ . However, SGLTs are dynamic proteins that undergo concerted conformational changes throughout sugar transport, following the “rocking bundle” principle. This inherent dynamism provides fertile ground for allosteric modulation. Allosteric modulators would bind to sites distinct from the primary substrate binding pocket, influencing SGLT1 function by inducing conformational changes and stabilising specific states (e.g., an occluded or inward-open state) that prevent glucose release or an outward-facing state that prevents binding without directly competing for the substrate.

Therefore, allosteric modulation targeting approach, particularly on the structural and mechanistic differences such as the EL5c loop, allows the scientific community to advance towards developing truly selective SGLT1 inhibitors, unlocking their full therapeutic potential.

## Conclusion

Selective inhibition of SGLT1 remains a compelling therapeutic opportunity across diabetes, cardiovascular disease, and cancer. By integrating cryo-EM structures with AlphaFold-assisted modeling, MD simulations, 3D-RISM water analysis, and binding/uptake assays, this review highlights where computational predictions are experimentally validated (e.g., outward-open ${Na}^{+}$ -bound vs. inward-open ${Na}^{+}$ free states; residue-level determinants) and where uncertainties persist (Na3 occupancy in SGLT2, MAP17 dependence, and water-path effects). A convergence workflow of high-resolution state determination, prospective mutagenesis, energetic mapping, and iterative medicinal chemistry supported by AI and machine learning conformational analysis offers a practical route to mechanism-driven design. Together, these strategies can refine current models of sodium-glucose transport and accelerate truly selective SGLT1 inhibitors. Therefore, approaches for achieving SGLT1 selectivity include prioritizing Na3 binding site engagement, leveraging EL5c-specific polar contacts, and filling SGLT1’s larger central pocket while avoiding SGLT2-favored hydrophobics and monitoring TM10/TM1 water-path occlusion in gut-restricted scaffolds. To fully understand the binding interactions of SGLT inhibitors, further studies with advanced techniques such as Cryo-EM and MD simulations are crucial to fully understand unresolved specific binding modes such as those of O- and N-glucosides inhibitors.
